# Design and validation of the psychosexual harassment questionnaire

**DOI:** 10.5249/jivr.v15i1.1777

**Published:** 2023-01

**Authors:** Sayed Ali Sharifi Fard, Fazlollah Hasanvand, Mohammad Ahmadpanah, Mohammad Reza Zoghi Paidar, Zahra Kazemi, Mahmoud Parchami Khorram

**Affiliations:** ^*a*^ Faculty of Educational Sciences and Psychology, University of Mohaghegh Ardabili, Ardabil, Iran.; ^*b*^ Faculty of Educational Sciences and Psychology, Allameh Tabataba'i University, Tehran, Iran.; ^*c*^ Department of Clinical Psychology, School of Medicine, Hamadan University of Medical Sciences, Hamadan, Iran.; ^*d*^ Behavioral Disorders and Substances Abuse Research Center, Hamadan University of Medical Sciences, Hamadan, Iran.; ^*e*^ Department of Psychology, Faculty of Economics and Social Sciences, Bu Ali Sina University, Hamadan, Iran.; ^*f*^ Faculty of Educational Sciences and Psychology, Shahid Beheshti University, Tehran, Iran.

**Keywords:** Psychological Measures, Physical Harassment, Sexual Harassment, Verbal Harassment, Virtual Harassment

## Abstract

**Background:**

Physical and sexual harassment has extensive psychological consequences on people's lives. Therefore, the using of a valid measure to identify this unpleasant experience in people can be useful both in determining the starting point of interventions related to victims and in general screenings in the society. In this regard, due to the lack of native and multidimensional measures to investigate this phenomenon, the aim of this study was to design and validation of the psychosexual harassment questionnaire.

**Methods:**

The research method was applied in terms of purpose and descriptive in terms of nature. The study population included all university students aged 18 to 30 in Hamadan province from 2021-2022. From this population, a sample of 600 participants was selected based on a multi-stage cluster sampling method according to the population of the studied cities. The measures were a 27-item researcher-made psychosexual harassment questionnaire and the Ryff Psychological Well-being Scale.

**Results:**

The results showed that the factor load was 27 items appropriate and 2 items inappropriate which were removed from the questionnaire. Finally, four factors including sexual harassment, physical harassment, sexual-virtual harassment, and verbal harassment were identified, in total, four factors could explain 58% of the variance of psychosexual harassment. Based on this, the four identified factors explained 33, 12, 8, and 5 percent of the variance of the structure of the psychosexual harassment construct, respectively. The adequacy of Kaiser-Meyer-Olkin sampling and Bartlett sphericity test (7332.2132) was calculated to be significant at the level of 0.001. The overall reliability of this questionnaire was calculated based on Cronbach's alpha coefficient equal to 0.91 and the reliability of physical, sexual, sexual-virtual and verbal harassment dimensions equal to 0.90, 0.88, 0.81, and 0.82, respectively.

**Conclusions:**

As a result, given the validity and reliability of this measure, researchers can use this measure to determine the level of four cases of abuse expressed. Also, due to having a nominal table and its interaction with each of the four dimensions of the measure, followed by obtaining very accurate and detailed information from the subject, clinicians can use this measure for clients and patients, especially in the category of disorders.

## Introduction

Security is the cornerstone of shaping the desire of individuals to actively and enthusiastically participate in a variety of social environments. Women and men engage in positive social interactions only if they are confident of their physical and mental health in predictable contexts and away from threatening factors. In the meantime, due to the high frequency of sexual, physical, and verbal abuse, especially among children, women, and girls.^1-2^ subsequently, more behavioral and emotional problems and disorders have been reported in these groups.^1-3^ In other words, sexual harassment is one of the factors that has become increasingly widespread all over the world and in different societies and has threatened all members of society, especially children and adolescents.^4-5^ Unstable working conditions, hierarchical organizations, the normalization of gender-based violence, toxic patriarchy, a culture of silence, and lack of leadership and active management in crime control are some of the key features that have made sexual harassment possible.^6^

Due to the wide scope of this concept, it is challenging and difficult to reach a comprehensive definition.^7^ Hence, different definitions have been given. In the most common descriptions, sexual harassment is defined as a gender-centered differentiation that creates a hostile educational and professional environment and, for the victim, many serious harms to work or the ability to participate in education, Job, and social.^8-9^ Sexual harassment is also part of the continuum of various actual and potential manifestations of gender-based violence in human systems (such as the higher education system), ranging from coercion and sexual language to sexual abuse and rape.^10^ This concept has some overlap with the concept of child sexual abuse; which means any sexual encounter of children with an adult.^11^ It should be noted to understand the difference between the two concepts, that research on child sexual abuse has often focused on victims' extreme experiences and acts of sexual or physical contact or influence,^8^ while sexual harassment also includes verbal and non-verbal communication with unwanted sexual content.

The various classifications made by theorists focus on different aspects of sexual harassment. Dziech and Weiner refer to public and private harassment.^12^ Miller^13^ in another theory to four classes of abusers including hunter (humiliating other people in behaviors such as sexual extortion), domineering (behavior to satisfy the next dimension of my personality), strategic or territory-oriented (trying to Protecting your special rights in the workplace or unique places) and on the street (harassment outside the home and work environment). In Baker ‘s classification, the categories of sexual harassment also include Power actors (sexual benefits of senior individuals from subordinates), Guidance (creating a supportive and guiding relationship with their victims and using this role to cover up their sexual intent and interaction), bullying (embarrassing with erotic comments), serial bullies (creating a positive image of oneself in others for sexual goals), physical abusers (unwanted physical contact), situational abusers (using people exposed to stressful life situations), High-profile bullies (making overly private comments), and embarrassing (calling women by their first name).^14^ Heslop explains sexual harassment based on two specific environments: hostile environments (any actions or comments about the victim's race, religion, etc., in the form of sexually harassing behaviors) and exchange environments (exchange of sexual interests for personal demands). In the theory of Buchanan, Bluestein, Nappa, Woods & Depatie, sexual harassment is divided into three categories: gender harassment, unwelcome sexual attention, and sexual coercion.^15^

which may be because of the presence of these people in social networks and messengers to the title of places to experience sexual harassment has increased.^20-21-22^ In connection with the second point, it should be acknowledged that sexual harassment causes symptoms of various emotional and behavioral disorders.^1-3-23^ Psychological and physical consequences of physical abuse for victims include depression, anxiety, low self-esteem, substance abuse, sexual dysfunction, functional gastrointestinal disorders, headache, chronic pain, and physical somatic symptoms.^24-25-26^ Therefore, due to the very high prevalence of violent crime and sexual and physical harassment among students in Iran,^27-28^ Clinical attention, accurate epidemiology followed by prevention and education are important. In this regard, one of the essential measures to eradicate sexual and physical harassment is to build measures to identify the extent of the epidemic and describe how this harmful phenomenon is in society. Because it provides a basis for raising awareness and planning for appropriate actions.

The high rate of abusive behavior and the adverse consequences of this phenomenon prove the importance of research in this field.^1-16^ Although it is challenging to obtain statistics on the level of sexual harassment in which young people are sacrificed, research has shown that sexual harassment is accompanied by aggression and severe bullying, it has been the biggest problem for women politicians around the world.^2^ Women in college and homosexuals and bisexuals have reported high levels of sexual harassment.^17^ The prevalence of this phenomenon in girls' schools is also very significant,^18-19^

In this regard, in relation to psychological-sexual harassment, various measures have been made, including: the Emotional Abuse Questionnaire (EAQ) consisting of six subscales (verbal abuse, emotional rejection, over control, inadequate control, high expectations, intimidation);^29^ The Self-Reporting Scale for Child Abuse (CASRS) in Iranian students based on the four dimensions of neglect, sexual, physical and emotional abuse;^30^ sexual harassment questionnaire based on the components of unwanted sexual attention, sexual harassment and sexual coercion;^31^ sexual and physical harassment questionnaire based on two-dimensional structure, visual, verbal and physical dimensions;^32^ Verbal Abuse Questionnaire in Korea (K-VAQ) in connection with verbal aggression;^33^ Scale of Internet Use for Sexual Purposes Based on the three components of explicit sexual observation, sexual partner search, and sexual information search;^34^ workplace harassment questionnaire (EAPA-T) based on four components (self-control in the workplace, emotional abuse, job discredit and devaluation of their role in the organization,^35^ small-scale measurement of the psychological climate underlying sexual harassment.^36^ Overall, it can be concluded that sexual harassment, significantly as a common theme worldwide, has profoundly affected individuals, groups, and organizations and has gained particular importance in recent decades. And as a complex issue due to the lack of consensus on accurate definition and measurement, it faces shortcomings in the construction of related measures and therapies.^31^ Studies show that due to the lack of reliable measures, the design of new, native, accurate, and multidimensional measures in this field seems necessary. Accordingly, a psychosexual harassment questionnaire has been designed, constructed, and validated in the present study.

## Methods 

This research was applied in terms of purpose and descriptive in nature. The study population included young students in university aged 18 to 30 years in Hamadan province from 2021-2022. The sampling method was relatively stratified according to the population of the studied cities based on the multi-stage cluster method. 

Due to the sensitivity of the research and incomplete completion of the questionnaire or the existence of lost data and possible discarding by some participants, a sample of 600 people was selected and due to provincial research, first purposefully in four cities (to cover different ethnicities), and then the link to the questionnaire was provided to the participants online. According to the Mueller method, the minimum sample required in this type of study is 5 and the maximum is 20. In this study, an attempt was made to collect the maximum size. Based on this, 600 questionnaires were distributed, and finally, after removing the undesirable data, the ratio of the number of samples to the number of questions was calculated as 1:19. It is worth mentioning that in the data purification in the manual stage, 40 participants and in the software purification stage, 50 participants were identified as incomplete data and outliers and were excluded from the analysis. 

Inclusion criteria of participants include being in the age range of 18 to 30 years, voluntary cooperation, ability to communicate, familiarity with Persian language, living in the target cities, and exclusion criteria include lack of proper cooperation of participants, having a mental illness, and obtaining invalid and incomplete data.

The present paper of the research project with the number: 9910307584 and Code of Ethics Committee: IR.UMSHA.REC.1399.792 It should be noted that the research project is related to Hamadan University of Medical Sciences in Iran.

**Measures**

*Psychosexual Harassment Questionnaire (PSHQ-27):*The researcher-made sexual harassment questionnaire has 27 items used to assess the extent of physical, sexual, verbal, and sexual-virtual (sexual) harassment. The measure also includes a descriptive table that can provide accurate and comprehensive information in interaction with four dimensions. The scoring scale of this measure is based on a six-point Likert scale from usually (6) to never (1). A higher score indicates more significant exposure to psychological sexual- harassment. In addition to utilizing content validity based on the corrective opinions of experts, the psychometric properties of other measures, including reliability and criterion validity, will also be examined. (Appendices A &B) 

**Psychosexual Harassment Questionnaire (PSHQ-27):** The following questionnaire has been prepared with the aim of measuring psychological (including physical and verbal) and sexual (including sexual-physical and sexual-virtual) harassment. This questionnaire has a quantitative dimension (rated on a six-point scale) and a qualitative dimension (supplementary quantitative and demographic questions). Therefore, you can choose one option in response to quantitative questions in Appendix A (1 to 27) and one or more options in response to supplementary and demographic questions in Appendix B (1 to 6).

Appendix A.							
Dimensions	Items	Frequency					
		Usually	Frequently	Sometimes	Occasionally	Rarely	Never
Physical harassment	1- Have you ever been beaten by someone (Such as kicking, slapping, squeezing the throat or similar things)?						
	2- Has this beating been so severe that a part of your body being red or bruised?						
	3- Have you ever been beaten by someone with an object such as a belt or a knife, or by throwing an object?						
	4- Has this beating - with or without a physical object - been enough to burn, tear or break any part of your body?						
	5- Have you ever been physically punished due to forcing others to do a request, and not doing it?						
	6- Have you been physically punished as a child for not following rules and regulations?						
Sexual harassment	7- Have you ever encountered sexual behavior (Such as touching or contacting body parts) in public places, against your will?						
	8- Have you ever experienced sexual looks from others, which lead to your harassment?						
	9- Have you ever been sexually caressed by someone against your will? (Such as being kissed and touched, with sexual intent)						
	10- Have you ever been forced to perform a certain sexual behavior by someone, with threats?						
	11- Have you ever been in a situation, where someone tries to touch your private parts against your will?						
	12- Has anyone ever forced you to have sex against your will?						
	13- Was this sexual intercourse, an oral sex?						
	14- Was this sexual intercourse, an anal sex?						
	15- Was this sexual intercourse, a vaginal sex?						
	16- Was this sexual intercourse something other than items 13, 14 and 15?						
Sexual-virtual harassment	17- Have you ever been threatened by someone to reveal sex through sharing private photos or videos?						
	18- Have you ever received a photo or video with sexual content from someone against your will?						
	19- Have you ever received text messages containing sexual content against your will?						
	20- Have you ever been invited to have sex against your will by someone on social network platforms?						
	21- Does reading inappropriate sexual texts in online discussion groups harass you?						
Verbal harassment	22- Have you ever heard sexual phrases from someone against your will?						
	23- Have you ever faced ugly curses from someone (family or others)?						
	24- When you were a child or teenager, was the way family members spoke to you in such a way that you felt weak or inferior?						
	25- Have you ever been addressed by someone with inappropriate titles and terms against your will?						
	26- Have you ever been harassed by hearing shouts containing nasty phrases from close people (e.g., family members)?						
	27- Have you ever been harassed by hearing sexual taunts or obscene swearing in public places?						

**Appendix B.** Appendix B.

Items						
1-Who has threatened or physically harassed you?	Family member	Close relative	Same-sex friend	Non-same-sex friend	Stranger	Nobody
2- At what age or ages have you been threatened or physically harassed?	Less than 6 years	Between 6 to 12 years	Between 12 to 18 years	Between 18 to 24 years	More than 24 years	Never
3-With which person or persons have you talked about threats or physical harassment?	Relatives	Family member	Close friends	Psychologist	Teacher or professor	Nobody
4-Who has sexually harassed you?	Family member	Close relative	Same-sex friend	Non-same-sex friend	Stranger	Nobody
5- At what age or ages have you been threatened or sexually harassed?	Less than 6 years	Between 6 to 12 years	Between 12 to 18 years	Between 18 to 24 years	More than 24 years	Never
6- Which person or people have you talked to, about the sexual harassment you experienced?	Relatives	Family member	Close friends	Psychologist	Teacher or professor	Nobody
7-Through which methods have you been trained to prevent sexual harassment?	Parents	Sister / Brother	Social media	Close friends	School / University	I have not seen training
8-Have some of the people around you experienced sexual or physical harassment?	Parents	Sister / Brother	Close friends	Relatives	Neighbor	Nobody

*Ryff Psychological Well-being Scale (RSPWB-18):*This scale was developed by Ryff (1989)^37^ and revised Ryff (2002).^38^ This scale has six components: independence, mastery of the environment, personal growth, positive communication with others, purposefulness in life, and acceptance. The sum of the scores of these six components is calculated as the total score of psychological well-being. This test is answered in a 6-point continuum from strongly disagree (1) to strongly agree (6). A student sample has standardized this version by Khanjani et al., in Iran, and an excellent internal consistency has been obtained according to Cronbach's alpha.^39^ The coefficients obtained for the six independence components are 72%, mastery of the environment, 76%, personal growth, 73%, positive communication with others, 75%, purposefulness in life, 52%, self-acceptance, 51%, and 71% for the whole scale. Tables were used to describe the research sample, and to show the information on demographic variables, and descriptive indicators were used to clarify the research variables. Also, in the inferential section, the structure and dimensions of the researcher-made questionnaire were discovered using exploratory factor analysis in the first step. In the confirmatory factor analysis section, the validity of the questionnaire structure was examined using first and second-order analysis. Data analysis in this study was performed using SPSS and LISREL software.

## Results

After deleting incomplete data and outliers, 510 participants remained for further analysis. 

Table 1 : Descriptive findings showed that the age of the participants at the time of exposure to physical harassment indicates that 10 participants (2%) did not report any case of physical harassment. Among them, 45 participants (8.8%) were under six years old, 165 (32.4 %) between 6 to 12 years old, 187 (36.7%) between 13 to 18 years old, 66 people (12.8 %) between 19 to 24 years, and 37 (7.3 %) over 24 years have been exposed to some form of physical harassment (From rarely to usually). In the findings related to sexual harassment, 333 participants (65.3 %) did not have experience with sexual harassment. 29 (5.7%) under 6 years old, 53 (10.3%) between 6 to 12 years old, 46 (9%) between 13 to 18 years old, 37 (7.3%) between 19 to 24 years old, and 12 (2.4%) have sexually harassed over 24 years old. 

**Table 1:** Descriptive Statistics.

Variable	Variable levels	Frequency (N)	Percentage (%)
**Age of physical harassment**	Never	10	2
	Less than 6 years	45	8.8
	Between 6 to 12 years	165	32.4
	Between 13 to 18 years	187	36.7
	Between 19 to 24 years	66	12.8
	More than 24 years	37	7.3
**Age of sexual harassment**	Never	333	65.3
	Less than 6 years	29	5.7
	Between 6 to 12 years	53	10.3
	Between 13 to 18 years	46	9
	Between 19 to 24 years	37	7.3
	More than 24 years	12	2.4
**Perpetrator to physical harassment**	Nobody	10	2
	Stranger	238	46.7
	Non-same-sex friend	30	5.9
	Same-sex friend	129	25.3
	Close relative	38	7.5
	Family members	65	12.8
**Perpetrator to sexual harassment**	Nobody	333	65.3
	Stranger	55	10.8
	Non-same-sex friend	45	8.8
	Same-sex friend	18	3.5
	Close relative	47	0.2
	Family members	12	2.4
**Trusted person for talking about physical harassment**	Nobody	393	77.1
	Teacher or professor	8	1.6
	Psychologist	18	3.5
	Close friends	62	12.2
	Relatives	26	5.1
	Family members	3	0.6
**Trusted person for talking about sexual harassment**	Nobody	374	73.3
	Teacher or professor	7	1.4
	Psychologist	20	3.9
	Close friends	75	14.7
	Relatives	29	5.7
	Family members	5	1
**Knowledge about the ways of prevent sexual harassment**	I have not seen training	208	40.8
	School / University	74	14.5
	Close friends	56	11
	Social media	115	22.5
	Sister / Brother	7	1.4
	Parents	50	9.8

In relation to the perpetrator of physical harassment, 10 (2%) participants did not report physical harassment, 238 (46.7 %) by a stranger, 30 (5.9%) by a non-same-sex friend, 129 (25.3%) by a same-sex friend, 38 (7.5%) by a close relative and 65 (12.8%) have been physically harassed by family members (From rarely to usually). Regarding the perpetrator of sexually harassing, 333 (65.3%) did not experience sexual harassment, 55 (10.8%) by a stranger, 45 (8.8%) by a non-same-sex friend, 18 (3.5%) by a same-sex friend, 47 (9.2%) by a close relative and 12 (2.4%) participants by family members (From rarely to usually). 

About the trusted person for talking about physical harassment, 393 (77.1%) participants did not attempt to talk about the harassment with others, 8 participants (1.6%) talked to their teacher or professor, and 18 (3.5%) with a psychologist, 62 (12.2%) with close friends, 26 (5.1%) with a close relative and 3 (0.6%) participants with family members. Also, about sexual harassment, 374 (73.3%) participants did not talk about harassment with others, 7 (1.4%) talked to their teacher or professor, and 20 (3.9%) with a psychologist. 75 (14.7%) with close friends, 29 (5.7%) with family members, and 5 participants (1%) with family members. Also, regarding the knowledge of ways to prevent sexual harassment, 208 (40.8%) participants did not receive training, 74 (14.5%) receive training from school or university, 56 (11%) from close friends, 115 (22.5%) from the social media, 7 (1.4%) from sister or brother and 50 (9.8%) participants from parents. 

Also, regarding the amount of psycho-sexual harassment in this study, 20.6% of participants experienced rare harassment, 34.1% experienced occasional harassment, 21.8% experienced sometimes harassment, and 23.5% experienced frequent harassment. About the sexual dimension, 333 (65.3%) participants experienced rare harassment, 77 (15.1%) had occasional harassment, 51 (10%) had sometimes harassment, and 49 (9.6%) had frequent harassment. About the physical dimension, 10 (2%) participants experienced rare harassment, 223 (43.7%) had occasional harassment, 151 (29.6%) had sometimes harassment, and 126 (24.7%) had frequent harassment. About the verbal dimension, 196 (38.4%) participants experienced rare harassment, 88 (17.3%) had occasional harassment, 92 (18%) had sometimes harassment, and 134 (26.3%) had frequent harassment. About the sexual-virtual dimension, 220 (43.1%) participants experienced rare harassment, 108 (21.2%) had occasional harassment, 100 (19.6%) had sometimes harassment, and 82 (16.1%) had frequent harassment. 

**Validity of the questionnaire**

A) Content validity: Due to the complex nature of psychosexual harassment and the expansion of the roots of this phenomenon in various disciplines (sociology, psychology, and other humanities) and little research history about it in the scope of psychology in Iran, the construction of measures was done with outstanding research. In the first step, an in-depth study was conducted based on the sources in the sexual and physical harassment field, and the necessary background information was provided to write the primary draft of the psychological-physical abuse questionnaire. Then, the questionnaire with 37 items was given to the experts to express their corrective opinions. Following this step, the necessary changes were applied by removing 7 items in the questionnaire and the ambiguities of the experts were resolved. In the first executive step, the introductory form of the questionnaire was given to a group of students, and they were asked to read the items carefully and identify ambiguous words and phrases in a column (1 ambiguous item was removed). Finally, after modifying the questionnaire based on the ambiguities raised by the students, the final form was prepared with 29 items and implemented.

B) Criterion validity: To evaluate the criterion validity, the correlation between this questionnaire and Ryff Psychological Well-Being Scale^40^ was used; The Pearson correlation coefficient between the broad psychosexual harassment questionnaire and psychological well-being was equal to 0.41, which indicates the validity of the criterion of the physical and sexual abuse questionnaire. Also, the correlation between sexual, physical, verbal, and sexual-virtual harassment dimensions with well-being was -0.47, -0.41, -0.35, and -0.29, respectively.

C) Structural validity: The structural validity of this questionnaire was confirmed using the statistical methods of exploratory factor analysis and confirmatory factor analysis. Also, using second-order factor analysis, each factor of the questionnaire on the formation of the structure of psycho-sexual harassment was investigated. Exploratory factor analysis: Exploratory analysis of the questionnaire was performed based on 29 items. Preliminary results of the analysis of the main components (items) showed that except for two items, the others had a suitable factor load. Accordingly, the adequacy of Kaiser-Meyer-Olkin (KMO) sampling and Bartlett sphericity test (7332.2132) was calculated to be significant at the level of 0.001. Due to the high level of these two tests, the correlation matrix was suitable for factor analysis.

According to Table 2 , eigenvalue values and percentage of variance explained by each of the identified factors in the psychosexual harassment questionnaire are reported. according to this, principal component analysis and orthogonal rotation of the Varimax type were used for factor analysis. Preliminary statistical characteristics by performing principal component analysis showed that the eigenvalues of four factors are more significant than one, and these four factors explain 58% of the total variance of the psycho-sexual harassment variable. Based on this, the four identified factors explained 33, 12, 8, and 5 percent of the variance of the structure of the psychosexual harassment construct, respectively.

**Table 2:** Eigenvalues and variance percentage explained by factors identified in psychosexual harassment questionnaire.

	eigenvalue	Percentage of variance	Cumulative percentage
1	8.795	32.572	32.572
2	3.207	11.876	44.448
3	2.257	8.358	52.806
4	1.389	5.145	57.952

According to Table 3 , the four identified factors had 10, 6, 6, and 5 items with appropriate factor load, respectively. Accordingly, the first factor, sexual harassment, accounted for about 33% of the total variance of psychosexual harassment, and the other three factors explained about 12, 8, and 5% of the variance, respectively. Overall, the identified factors could predict 58% of the variance of the variable of psychosexual harassment.

**Table 3:** Rotated correlation matrix.

Factors Items	Extracted agents			
	Sexual harassment	Physical harassment	Verbal harassment	Sexual-virtual harassment
Item1	0.41			
Item2	0.60			
Item3	0.62			
Item4	0.76			
Item5	0.81			
Item6	0.73			
Item7	0.77			
Item8	0.71			
Item9	0.74			
Item10	0.58			
Item1		0.81		
Item2		0.84		
Item3		0.86		
Item4		0.80		
Item5		0.74		
Item6		0.62		
Item1			0.40	
Item2			0.67	
Item3			0.70	
Item4			0.69	
Item5			0.74	
Item6			0.62	
Item1				0.44
Item2				0.77
Item3				0.82
Item4				0.66
Item5				0.42

**First-order confirmatory factor analysis**

**Figure 1:**
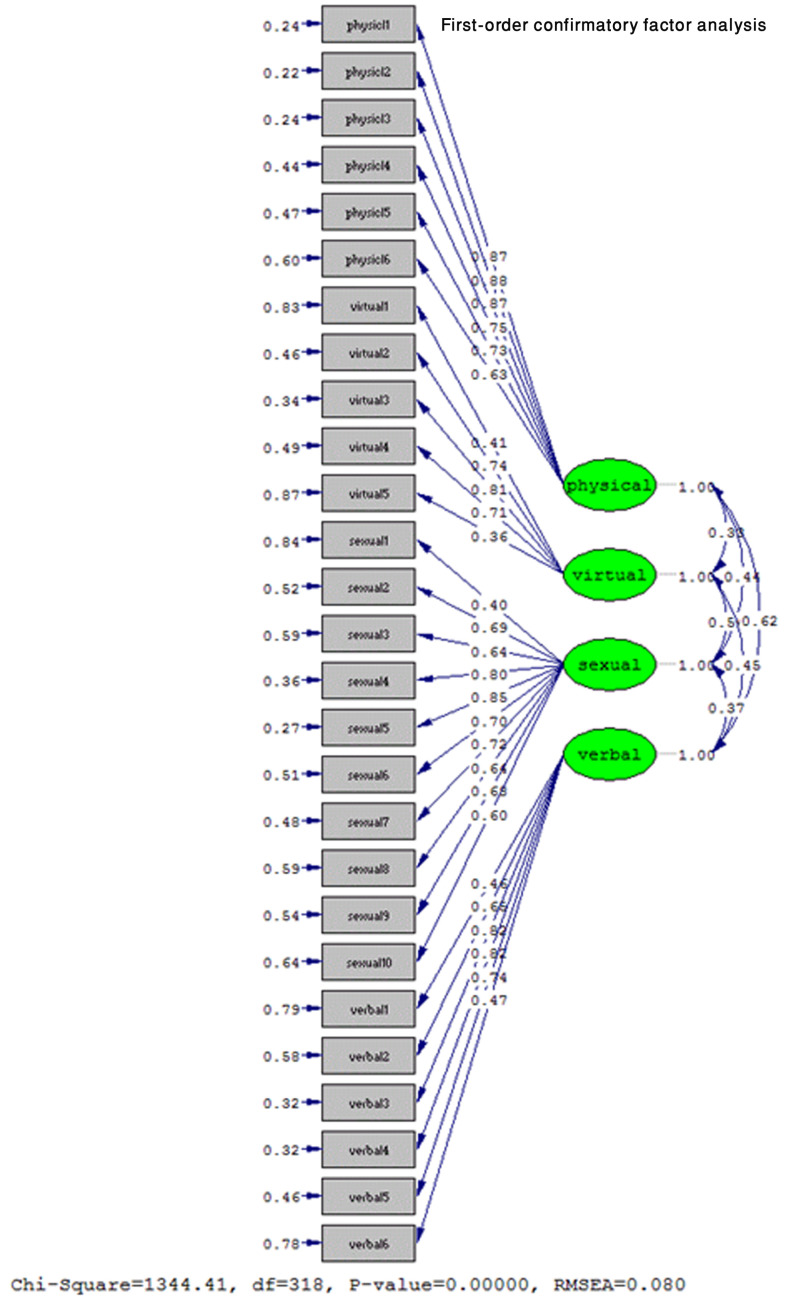
Confirmatory factor analysis model of psychosexual harassment in standard coefficients.

As shown in Figure 1, in the fitted model, a psychosexual harassment questionnaire in the mode of coefficients has been standardized to confirm the confirmatory factor. The factor load of all items is calculated above 0.36, and thus, the results of exploratory factor analysis are confirmed.

**Figure 2:**
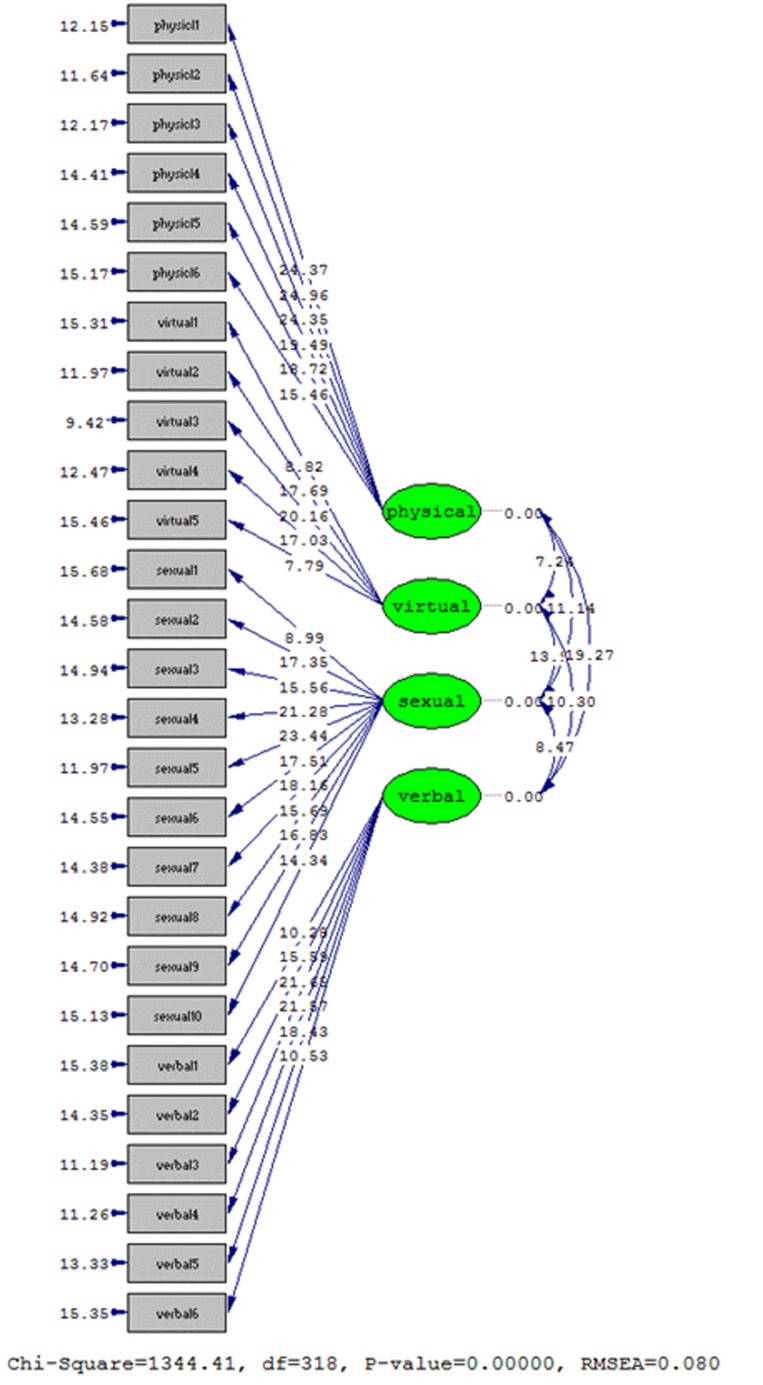
Confirmatory factor analysis model of psychosexual harassment in the case of significant coefficients.

As can be seen in Figure 2, since the calculated t-values for the questionnaire items are higher than 1.96, the questions have the necessary validity and the validity of attending the questionnaire.^41^ The findings of confirmatory factor analysis confirm the results of the exploratory factor analysis that the four-factor structure of psycho-sexual harassment is. In analyzing structural equations, acceptable scientific criteria for confirming the theoretical model using the collected data is the main discussion in the "model fit indices." The root means the square index of estimation errors was confirmed as a basic fit index of the model if it was less than 0.1 level and in this model was equal to 0.80. In addition, when the result of dividing the chi-square factor by df is less than 5, the model's fit is confirmed in the appropriate state, and this index in the present study was calculated to be 4.22, which indicates the proper fit of the model. Is. Also, when the standard fit indices, non-normalized fit, adaptive fit, this deafness fit, relative value, and goodness of fit higher or equal to 0.9 are calculated, the appropriate fit of the model is confirmed, and this research, this statement is true. Because these indices were equal to 0.93, 0.94, 0.95, 0.95, 0.93, and 0.91, respectively.

**Second-order confirmatory factor analysis**

**Figure 3:**
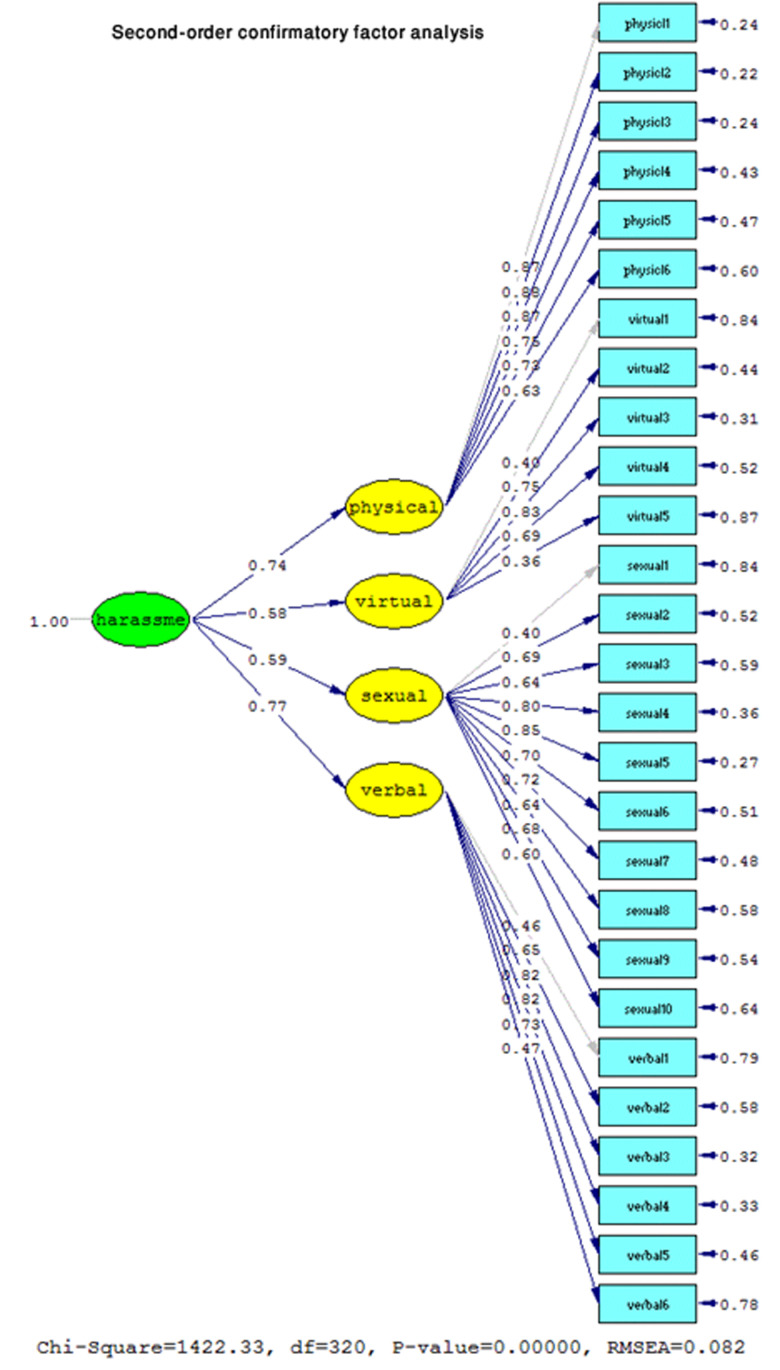
Confirmatory factor analysis of the second level of psychosexual harassment questionnaire in the form of standard coefficients.

As shown in Figure 3, in the fitted model of the psychosexual harassment questionnaire, to analyze the second-order confirmatory factor, the standard coefficients of all four factors have been calculated to an appropriate extent.

**Figure 4:**
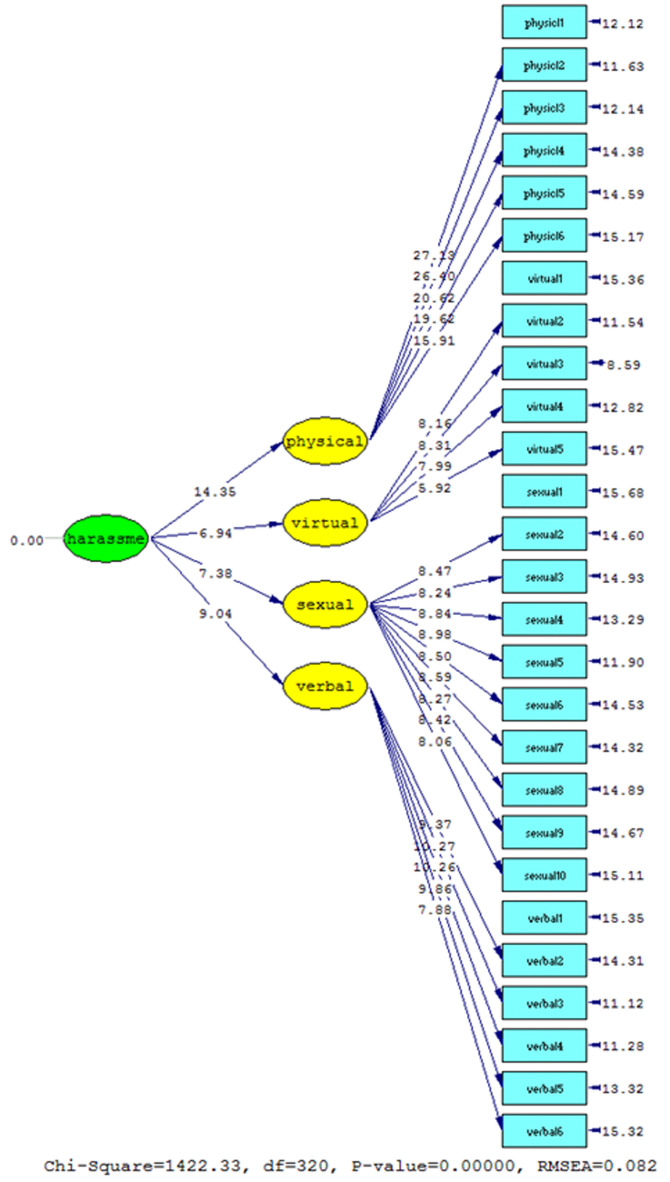
Confirmatory factor analysis of the second level of psychosexual harassment questionnaire in the case of significant coefficients.

According to Figure 4, considering that the t-value calculated for the four factors of the psychosexual harassment questionnaire is higher than 1.96, all the factors in the formation of psychosexual harassment have the necessary validity and have a significant nature in the questionnaire. Accordingly, the second-order confirmatory factor analysis findings confirm the results of the exploratory factor analysis that the structure is four-factor and based on the standard impact coefficients, verbal, physical, sexual, and sexual-virtual dimensions, respectively 0.77, 0.74, 0.59, and 0.58 with t values equal to 14.35, 9.04, 7.38, and 6.94 have a significant role in forming the concept of psychosexual harassment. In the fitting study of this model, it was found that the root means square of the estimation errors is equal to 0.082, which was less than 0.1, and was confirmed. The result of dividing the chi-square factor by df was calculated to be less than 5 (4.44), which is appropriate. Also, since the calculated statistic for other indicators was higher than 0.9, the appropriate fit of the model was confirmed.

**Reliability**

Cronbach's alpha coefficient was used to evaluate the reliability of the researcher-made questionnaire. Accordingly, the overall reliability of the questionnaire was 0.91, and the reliability of the dimensions of sexual harassment (10 items), physical harassment (6 items), verbal harassment (6 items), and sexual-virtual harassment (5 items) was 0.90, 0.88, 0.81, and 0.82 calculated, respectively, which indicates the overall reliability of the model and its dimensions.

**Test norm**

Table 4 shows that the test's norm is based on the T scale with a mean of 50 and a standard deviation of 10, obtained from the following formula. In this formula, the balanced score T is equal to the Z scores extracted from the normal distribution table based on the raw scores.

**Table 4:** Test norm based on T and Z scores.

Raw score	Z	T	Raw score	Z	T	Raw score	Z	T
28	-1.21	38	56	0.52	55	84	2.25	72
30	-1.09	39	58	0.64	56	86	2.38	74
32	-0.97	40	60	0.77	58	88	2.50	75
34	-0.84	42	62	0.89	59	90	2.62	76
36	-0.72	43	64	1.01	60	92	2.75	77
38	-0.60	44	66	1.13	61	94	2.86	79
40	-0.47	45	68	1.26	63	96	2.99	80
42	-0.35	47	70	1.38	64	98	3.10	81
44	-0.22	48	72	1.50	65	100	3.21	82
46	-0.10	49	74	1.63	66	102	3.36	84
48	0.03	50	76	1.76	68	104	3.49	85
50	0.15	51	78	1.88	69	106	3.63	86
52	0.27	53	80	2	70	108	3.73	87
54	0.39	54	82	2.11	71	112	3.99	90
Mean of variable					Standard deviation			
Total scale			47.62		Total scale			16.14
Physical dimension			9.99		Physical dimension			5.28
Sexual dimension			13.93		Sexual dimension			5.58
Sexual-virtual dimension			9.01		Sexual-virtual dimension			3.93
Verbal dimension			14.68		Verbal dimension			6.34

**Test cut points**

The purpose of calculating the cut-off points is that after calculating his / her score in the psychological-sexual harassment test, each person can determine how he/she is in terms of being exposed to psycho-sexual harassment in society. For this purpose, four categories of harassment Based on the calculation of raw scores and T scores for the total scale and its dimensions were identified in Table 5. Accordingly, in the questionnaire's general level, the first category consisted of participants who had scored lower than 34 and, according to the levels of the questionnaire, were in the category of "no or rare harassment." According to the questionnaire levels, the second group consisted of participants with scores ranging from 35 to 44 and classified as "mildly harassment." The third group consisted of participants with a score of 45 to 56 and were classified as "moderate harassment" according to the questionnaire's levels. The fourth category consisted of participants with scores above 56 and was classified as "severely harassment" according to the questionnaire levels. Also, for all four dimensions of the questionnaire, all four levels - rare to severe - are specified in Table 5.

**Table 5:** Calculation of cutting points of the psychosexual harassment questionnaire based on the calculation of quartiles.

Dimensions of harassment	Cutting points based on T scores	Class title
Total questionnaire	Less than 42	Rare harassment
	Between 49 to 55	Mild harassment
	Between 49 to 55	Moderate harassment
	More than 55	Severe harassment
Sexual dimension	Less than 42	Rare harassment
	Between 42 to 48	Mild harassment
	Between 49 to 55	Moderate harassment
	More than 55	Severe harassment
Physical dimension	Less than 42	Rare harassment
	Between 49 to 55	Mild harassment
	Between 49 to 55	Moderate harassment
	More than 55	Severe harassment
Sexual-virtual dimension	Less than 42	Rare harassment
	Between 42 to 48	Mild harassment
	Between 49 to 55	Moderate harassment
	More than 55	Severe harassment
Verbal dimension	Less than 42	Rare harassment
	Between 49 to 55	Mild harassment
	Between 49 to 55	Moderate harassment
	More than 55	Severe harassment

## Discussion

The results obtained in the descriptive findings of the study indicate a 34.7% prevalence of sexual harassment and 98% of physical harassment. Also, according to the defined age ranges, physical and sexual harassment has a negative correlation with the age of individuals, so the main experiences of physical and sexual harassment have been at a young age, from under seven years old to 18 years old. Studies conducted inside and outside the country to investigate sexual and physical abuse prevalence have also yielded different results. In Riahi and Poor Almasi's research, 70% of women in the study reported sexual harassment experiences.^42^ In this study, sexual harassment experiences are negatively correlated with age. It is also observed in various studies 40% of sexual harassment,^43^ 61% experience harassment,^28^ 67.6% physical harassment, ^44^ 12.17% sexual harassment, and 7.38% physical harassment.^45^ The prevalence rate of reported harassment experiences in different age groups also shows different results. Also, in foreign studies, the rate of sexual harassment experiences of students has been reported as 40% to 85%.^46^

In Langer's study, sexual harassment was reported at 25 percent for women and 10 percent for men.^47^ Also, in terms of the age range of harassment experience, the findings of the researches in line with the findings of the present study, show that the highest rate of experience of bullying behaviors is between the ages of 12 and 16, and the lowest He was under 12 years old. The rate of experiencing abusive behaviors is 48.16% for men and 51.84% for women. This finding is consistent with the results of studies that show a higher rate of experiences of harassment of women than men.^47^ In this regard, men, and women often differ in what they interpret as sexual harassment. Most of those who reported sexual harassment were women, and most perpetrators were men.^48^ On the other hand, men are less likely to report their experiences of harassment.^49^ Third, abusive behaviors are more stressful for women than men.^50^

In the present study, more sexual, verbal, and sexual-virtual harassment was reported by women, which is consistent with the results of other studies.^32-50^ Physical abuse was more common in men than women, consistent with the results of other studies.^51-52-42^ Verbal harassment (29.63%) had the highest prevalence, and sexual-virtual harassment (18.74%) had the lowest prevalence. Many possible reasons justify the higher prevalence of verbal harassment. One of the reasons for this is social ugliness, and consequently, less public pressure to react to verbal harassment than sexual and physical harassment due to its high frequency and repetition, and cultural stereotypes make verbal harassment less critical. Also, the lack of objectivity and obviousness of verbal harassment compared to sexual and physical injuries can be another reason for the high levels of this type of harassment.

In the old studies, Higher levels of physical harassment in men are consistent with the results of research,^53^ in adolescence with the results of,^54^ and also.^55^ Higher physical abuse of men than women and a higher prevalence rate in adolescence may be influenced by cultural stereotypes and beliefs that men are more resistant than women and less vulnerable. Adolescent conflicts are due to various factors such as more independence at this age, differences in attitudes with adults and more expression by boys, and more common jokes and physical conflicts in It is among teenage boys.

Significant differences in the prevalence of sexual and physical harassment in different studies depend on various possible causes. One of the reasons is the different target populations in different researches. For example, studies that only looked at experiences of harassment in occupational situations such as hospital staff reported higher prevalence rates. It is natural that in environments such as hospitals, staff and employees interact more with people under stressful situations, which increases the rate of abusive behaviors, especially verbal harassment in these environments.

The most sexual and physical harassment by strangers (46.7% and 10.8%, respectively), the least sexual harassment by family members (2.4%), and the least physical harassment by non-same-sex friends (5.9%). These findings are consistent with the results of research showing that most sexual harassment is perpetrated by strangers^50-55^ but with the results of student research and Amini, in which the family did the most physical abuse, are different.

Descriptive findings related to the report of harassment experiences indicate that 77.1% of physical harassment and 73% of sexual harassment were not reported. These findings are especially important compared to another section of the 40.8% report of non-receipt of sexual harassment prevention training as part of the prevention of sexual harassment training. Sexual harassment includes instant information training when exposed to or experiencing harassment to prevent the perpetrator from continuing the harassing behavior and taking timely action to treat the physical and psychological harm inflicted. Also, considering the high prevalence of sexual and physical harassment according to the present study's findings, it can be said that these three findings are related to the high prevalence of harassment experiences, lack of high reporting of harassment experiences, and lack of higher education. Are together. In other words, not receiving training increases both harassment and harassment and is associated with not reporting harassment. Bullying training should focus on bullying prevention training and include people who are prone to bullying. For example, many parents' punitive and physically abusive behaviors toward their children are due to a lack of communication skills and a lack of knowledge about the specific characteristics of each stage of childhood and adolescence. On the other hand, non-reporting of harassment provides both safe and appropriate conditions for abusers and prevents the identification of abusers and victims from providing the necessary training in this area. Therefore, it can be expected that with the increase of necessary education related to the prevention of harassment or post-harassment education, the prevalence of sexual and physical harassment will have a decreasing trend. 

In the validity section, the criterion with the dual psychological welfare questionnaire was the overall correlation coefficient of -0.41. This finding is consistent with the results of studies that have examined the relationship between sexual and physical harassment and psychological well-being and show a significant relationship between harassment experiences and reduced psychological well-being.^19-56-57^ Abusive behaviors can affect different aspects of psychological well-being in different ways. For example, the experience of corporal and verbal punishment in relationships with others, and especially concerning parents, is likely to disrupt their acceptance process. Because on the one hand, people are usually punished for their negative characteristics and behavioral mistakes, on the other hand, as a general rule accepted in psychology, most of the attitudes of people about themselves are the same attitude. It has been received and internalized by those around them, especially parents. Therefore, a person with experiences of physical and verbal abuse cannot have a positive attitude toward himself and accept all the positive and negative aspects of himself. Also, a person suffering from sexual or physical harassment due to fear of others and creating an avoidant approach may not be able to establish good relationships and therefore directly and indirectly negatively affect his or her well-being. Of course, if the person is exposed to systematic and continuous harassment, relationship disorders and reduced well-being will become more severe. Also, all types of harassment can cause problems in the personal development component, which is an effective component in well-being, with harmful emotional and cognitive interactions. 

On the other hand, in harassment measurement measures, most studies without any theoretical framework rely on simple checklists with unknown reliability and validity.^58^ For some of these measures, unexpressed psychometric properties or expressed properties are limited. Others do not have the comprehensiveness to measure sexual harassment and cover only one aspect of sexual harassment. The number of measures measuring sexual harassment is limited to a specific situation, such as hospital staff or workplaces in general, and includes only harassment experienced over some time, such as the last 6 or 12 months. Some other scales are not specific measures for measuring sexual and physical harassment, but only a limited number of questions.

These measures question sexual and physical harassment. In this regard, the revised 9-item scale of sexual harassment and the 4-item scale of sexual coercion by Bendixen et al., report the Kuder-Richardson psychometric property for reliability.^45^ The Organizational Sexual Harassment Scale (OTSG) is also limited to measuring sexual harassment in an organization.^59^ The Pryor et al., Sexual Harassment Probability Scale (LSH) only measures men's likelihood of sexual harassment.^48^

Similarly, the Sexual Harassment Exposure Questionnaire (BSHS) measures sexual harassment in the workplace over the past six months.^59^ A revised version of the Sexual Harassment Assessment (AAUW) measures sexual harassment in adolescents.^60^ Although they include questions to assess sexual harassment, some measures are not explicitly designed to measure sexual harassment. For example, the Standard Workplace Violence Questionnaire (IL, WHO & ICN), designed by the International Labor Office, the World Health Organization, and the International Council of Nurses, has only three questions to estimate the prevalence of sexual harassment in the workplace.^61^ The 30-item Iranian scale of workplace harassment examines sexual harassment in the workplace without reporting the psychometric psychometrics information of the scale.^42^ With these interpretations, a few measures for measuring sexual harassment while reporting appropriate psychometric properties are more comprehensive than what was stated in measuring harassment behaviors.

For instance, the 19-item Sexual Harassment Questionnaire (SEQ) assessed the experience of sexual harassment in three subscales of sexual harassment, unwelcome sexual attention, and sexual coercion reported with appropriate psychometric properties.^58^ The SEQ is a questionnaire designed to measure only sexual harassment by teachers, classmates, and university staff over the past 12 months. Also, the mean SEQ scores are low and do not have a normal distribution.^62^ The Sexual Harassment Experiences Questionnaire for Pakistan Workplaces (SHSQ) is another questionnaire that has appropriate psychometric properties and comprehensiveness and is designed and constructed based on the SEQ questionnaire.^63^ This questionnaire was developed to measure sexual harassment in the workplace with 60 samples of working women. The results of standardization of this measure, due to the tiny number of samples used, the standardization of this measure cannot be highly generalizable and valid because according to the rules of psychometrics, construction and standardization of a questionnaire that has sufficient generalizability and validity, a large sample of people needs. In the present study, the sample consisted of 510 people to standardize the psycho-sexual abuse questionnaire.

Another important point about the psycho-sexual harassment questionnaire is to consider the sexual-virtual component. Nowadays, with the spread of electronic means of communication and the use of the Internet, primarily sexual-virtual social pages, many social interactions occur sexual-virtually in the form of social pages. Naturally, as with real-world interactions, some sexual-virtual actions and reactions are annoying. Although sexual-virtual interactions are only verbal and do not directly involve the behavioral aspect, according to the results of several studies that show the higher prevalence of verbal harassment and the importance and impact of this type of harassing behavior, in the psychological and sexual questionnaire of the present study, the component of sexual-virtual harassment is also included. Among the measures for measuring sexual harassment, some, such as Bendixen et al., have paid attention to this part of harassing interactions,^45^ but most of the mentioned measures, due to the history of designing and building old measures that sexual-virtual interactions are not widespread today. They lack a sexual-virtual harassment component.

## Conclusion

The results in the first part of this research showed that, in relation of perpetrator of physical harassment, strangers and same-sex friend had the highest frequency and in relation of perpetrator of sexual harassment, strangers and had the highest frequency, which shows that in order to prevent the occurrence of physical harassment, the manner of treating strangers and friends of the same age should be considered. In relation to trusted person for talking about physical and sexual harassment, close friends were identified as the most important trusted refuge. This means that people seek refuge in the close circle of non-relative acquaintances instead of going to specialists such as counseling and psychologists. In association with Knowledge about the ways of prevent sexual harassment, 208 did not receive any education, which is very worrying. And the most powerful way to receive information about the right ways to deal with harassment was social media, which is full of invalid and different information and is less reliable than sources such as psychologists and schools.

In connection with the main part of the research, psychosexual harassment questionnaire developed in the leading research, according to having the desired psychometric properties such as the number of questions, descriptive questions, subscales, method of implementation and scoring, how to calculate the total score, cut points as well as very good validity and reliability is a standard psychosexual harassment questionnaire. In the other word, due to its multidimensionality and variance explanation equal to 58%, this measure has a suitable comprehensiveness to be used in the field of screening a wide range of physical and sexual harassment, assessment of adverse sexual experiences and many related harms in this field. Therefore, after standardization in different communities and samples, in addition to research, this measure can be used to measure the amount of harassment in different dimensions according to its descriptive table for measurement in clinical interventions.

**Acknowledgements**

We appreciate and thank all the participants for their cooperation in completing this research project. We are also very grateful to Hamadan University of Medical Sciences Research Center for financial support.

## References

[1] Kaltiala-Heino R, Fröjd S, Marttunen M (2016). Sexual harassment victimization in adolescence: Associations with family background. Child Abuse Negl.

[2] Krook ML (2017). Violence against women in politics. Journal of Democracy.

[3] Bucchianeri MM, Eisenberg ME, Wall MM, Piran N, Neumark-Sztainer D (2014). Multiple types of harassment: Associations with emotional well-being and unhealthy behaviors in adolescents. J Adolesc Health.

[4] Mansell J, Moffit DM, Russ AC, Thorpe JN (2017). Sexual harassment training and reporting in athletic training students. Athletic Training Education Journal.

[5] Sakellari E, Berglund M, Santala E, Bacatum CM, Sousa JE, Aarnio H (2022). The Perceptions of Sexual Harassment among Adolescents of Four European Countries. Children (Basel)..

[6] Bondestam F, Lundqvist M (2020). Sexual harassment in higher education–a systematic review. European Journal of Higher Education.

[7] Pina A, Gannon TA, Saunders B (2009). An overview of the literature on sexual harassment: Perpetrator, theory, and treatment issues. Aggression and Violent Behavior.

[8] Stoltenborgh M, Van Ijzendoorn MH, Euser EM, Bakermans-Kranenburg MJ (2011). A global perspective on child sexual abuse: Meta-analysis of prevalence around the world. Child Maltreat.

[9] Gruber JE, Fineran S (2007). The impact of bullying and sexual harassment on middle and high school girls. Violence Against Women.

[10] Latcheva R (2017). Sexual harassment in the European Union: A pervasive but still hidden form of gender-based violence. J Interpers Violence.

[11] Senn TE, Carey MP, Vanable PA (2008). Childhood and adolescent sexual abuse and subsequent sexual risk behavior: Evidence from controlled studies, methodological critique, and suggestions for research. Clin Psychol Rev.

[12] Dziech BW, Weiner L. The Lecherous Professor. Sexual Harassment on Campus, 2nd ed. Baltimore: University of Illinois Press, 1990.

[13] Knudsen D. Abused and battered: Social and legal responses to family violence, 1st ed. New York: Routledge, 2017.

[14] Baker JA. The sexual harassment workbook: Recognizing, Preventing & Managing Sexual Harassment in the Workplace. 1st ed. Bayou Publishing LLC, 2015: 12-17.

[15] Buchanan NT, Bluestein BM, Nappa AC, Woods KC, Depatie MM (2013). Exploring gender differences in body image, eating pathology, and sexual harassment. Body Image.

[16] Chiodo D, Wolfe DA, Crooks C, Hughes R, Jaffe P (2009). Impact of sexual harassment victimization by peers on subsequent adolescent victimization and adjustment: A longitudinal study. J Adolesc Health.

[17] Pololi LH, Evans AT, Civian JT, Gibbs BK, Coplit LD, Gillum LH (2015). Faculty vitality—surviving the challenges facing academic health centers: a national survey of medical faculty. Acad Med.

[18] Dahlqvist HZ, Landstedt E, Young R, Gådin KG (2016). Dimensions of peer sexual harassment victimization and depressive symptoms in adolescence: A longitudinal cross-lagged study in a Swedish sample. J Youth Adolesc.

[19] Reed E, Salazar M, Agah N, Behar AI, Silverman JG, Walsh-Buhi E (2019). Experiencing sexual harassment by males and associated substance use & poor mental health outcomes among adolescent girls in the US. SSM Popul Health.

[20] Henry N, Powell A (2018). Technology-facilitated sexual violence: A literature review of empirical research. Trauma Violence Abuse.

[21] Stonard KE, Bowen E, Walker K, Price SA (2017). “They’ll always find a way to get to you”: Technology use in adolescent romantic relationships and its role in dating violence and abuse. Journal of Interpersonal Violence.

[22] Englander E (2015). Coerced sexting and revenge porn among teens. Bullying, Teen Aggression & Social Media.

[23] Sharififard SA, Boroujerdi M, Attaran E, Shast Fooladi M. Marriage without divorce 1st ed. Tehran: Danjeh Publications, 2019.

[24] Sadock BJ, Sadock VA, Ruiz P. Synopsis of psychiatry: Behavioral Sciences Clinical Psychiatry, 11th ed. New York: Wolters Kluwer, 2015.

[25] Houle JN, Staff J, Mortimer JT, Uggen C, Blackstone A (2011). The impact of sexual harassment on depressive symptoms during the early occupational career. Soc Ment Health.

[26] Willness CR, Steel P, Lee K (2007). A meta‐analysis of the antecedents and consequences of workplace sexual harassment. Personnel Psychology.

[27] Torkashvand F, Jafary F, Rezaeian M, Sheikh Fathollahi M (2013). A survey on child abuse and some demographic factors affecting students of the third grade of guidance school in Zanjan in 2011. Journal of Rafsanjan University of Medical Sciences.

[28] Daneshamouz B, Amini A (2005). Frequency of history of physical and sexual abuse in patients admitted to psychiatric wards. Cognitive Science News.

[29] Momtaz V, Mansor M, Talib MA, Kahar RB, Momtaz T (2022). Emotional Abuse Questionnaire (EAQ): A New Scale for Measuring Emotional Abuse and Psychological Maltreatment. Japanese Psychological Research.

[B30] Mohammadi MM, Mohammadi MR, Nazeri MA, Salavati MA, Razzaghi OM (2003). Development, validation, and reliability of child abuse self-report scale (CASRS) in Iranian students. Medical Journal of the Islamic Republic of Iran.

[31] Nielsen MB, Bjørkelo B, Notelaers G, Einarsen S (2010). Sexual harassment: Prevalence, outcomes, and gender differences assessed by three different estimation methods. Journal of Aggression, Maltreatment & Trauma.

[32] Vega-Gea  E, Ortega-Ruiz R, Sánchez V (2016). Peer sexual harassment in adolescence: Dimensions of the sexual harassment survey in boys and girls.. International Journal of Clinical and Health Psychology.

[33] Jeong B, Lee SW, Lee JS, Yoo JH, Kim KW, Cho S (2015). The psychometric properties of the Korean version of the verbal abuse questionnaire in university students. Psychiatry Investig.

[34] Velezmoro R, Negy C, Livia J (2012). Online sexual activity: Cross-national comparison between United States and Peruvian college students. Arch Sex Behav.

[35] Escartín J, Rodríguez-Carballeira Á, Gómez-Benito J, Zapf D (2010). Development and validation of the workplace bullying scale EAPA-T. International Journal of Clinical and Health Psychology.

[36] Estrada AX, Olson KJ, Harbke CR, Berggren AW (2011). Evaluating a brief scale measuring psychological climate for sexual harassment. Military Psychology.

[37] Ryff CD (1989). Happiness is everything, or is it? Explorations on the meaning of psychological well-being. Journal of Personality and Social Psychology.

[38] Ryff C, Singer B (2002). From social structure to biology. Handbook of positive psychology. Oxford University Press.

[39] Khanjani M, Shahidi S, Fath Abadi J, Mazaheri M, Shokri O (2014). Factor structure and psychometric properties of short form (18 questions) Ryff Psychological Well-Being scale in male and female students. Thought and Behavior in Clinical Psychology.

[40] Hooman HA. Structural equation modeling using LISREL software, 6th ed. Tehran: SAMT Publication, 2015.

[41] Riahi MI, Poralmasi Kh (2019). A comprehensive cognitive study of the experience of sexual harassment of women in the workplace. Social Welfare Research Quarterly.

[42] Sadeghi Fasaei S, RajabLarijani M (2011). A sociological study of sexual harassment of women in the workplace. Women in Development and Politics (Women's Research)..

[43] Zahrabi Moghadam J, Nouhjah S, Divdar M, Sedaghat Dyl Z, Adibpour M, Sephavand Z (2012). Frequency of child abuse and some related factors in 2-5 years children attending health centers of Ahvaz and Haftgel in 2011. Jentashapir Journal of Health Research.

[44] Rostami M, Abdi M, Heidari H (2014). Childhood abuse and the amount of forgivenessin married individuals. Thoughts and Behavior in Clinical Psychology.

[45] Bendixen M, Daveronis J, Kennair LE (2018). The effects of non-physical peer sexual harassment on high school students’ psychological well-being in Norway: Consistent and stable findings across studies. Int J Public Health.

[46] Cesario B, Parks-Stamm E, Turgut M (2018). Initial assessment of the psychometric properties of the Sexual Harassment Reporting Attitudes Scale. Cogent Psychology.

[47] Pryor JB, Giedd JL, Williams KB (1995). A social psychological model for predicting sexual harassment. Journal of Social Issues.

[48] Courtois CA, Spiegel J. Sexual abuse of males: The SAM model of theory and practice, 1st ed. New York, Routledge, 2003.

[49] Siller H, Tauber G, Komlenac N, Hochleitner M (2017). Gender differences and similarities in medical students’ experiences of mistreatment by various groups of perpetrators. BMC Med Educ.

[50] Rautio A, Sunnari V, Nuutinen M, Laitala M (2005). Mistreatment of university students most common during medical studies. BMC Med Educ.

[51] Lashkari K, Hashemi SZ (2015). Sociological study of sexual harassment of young women. Strategic Studies in Sports and Youth.

[52] Ross CA, Keyes BB, Xiao Z, Yan H, Wang Z, Zou Z (2005). Childhood physical and sexual abuse in China. J Child Sex Abus.

[53] Jones ED, McCurdy K (1992). The links between types of maltreatment and demographic characteristics of children. Child Abuse Negl.

[54] Macmillan R, Nierobisz A, Welsh S (2000). Experiencing the streets: Harassment and perceptions of safety among women. Journal of Research in Crime and Delinquency.

[55] Slaatten H, Anderssen N, Hetland J (2015). Gay related name‐calling among Norwegian adolescents–harmful and harmless. Scand J Psychol.

[56] Lichty LF, Campbell R (2012). Targets and witnesses: Middle school students’ sexual harassment experiences. The Journal of Early Adolescence.

[57] Fitzgerald LF, Gelfand MJ, Drasgow F (1995). Measuring sexual harassment: Theoretical and psychometric advances. Basic and Applied Social Psychology.

[58] Hulin CL, Fitzgerald LF, Drasgow F (1996). Organizational influences on sexual harassment (ed). Sexual harassment in the workplace: Perspectives, frontiers, and response strategies. Sage Publications.

[59] Einarsen S, Sørum DR (1996). "Arbeidskamerat eller sex-objekt?" En krysskulturell studie av seksuell trakassering i arbeidslivet. Nordisk Sexologi.

[601] Ortega R, Sánchez V, Ortega-Rivera  J, Nocentini A, Menesini E (2010). Peer sexual harassment in adolescent girls: A cross-national study (Spain-Italy). International Journal of Clinical and Health Psychology.

[61] Amini R, Mohammadi N, Karaji F, Tapak L (2020). Frequency of the type of workplace violence against Hamadan medical emergency technicians and its relationship with individual and occupational variables, 2018. Avicenna J Nurs Midwifery Care.

[62] Buchanan NT, Fitzgerald LF (2008). Effects of racial and sexual harassment on work and the psychological well-being of African American women. J Occup Health Psychol.

[63] Kamal A, Tariq N (1997). Sexual harassment experience questionnaire for workplaces of Pakistan: Development and validation. Pakistan Journal of Psychological Research.

